# 

**Published:** 1878-12

**Authors:** 


### CONTENTS:

To our Readers and Correspondents... 561

Original Lecture:
Hernia of the Cornea—Action of Eserine and Pilocarpine. By F. C. Hotz, M. D. 564

Original Communications:
Sequel and Fatal Termination of a Case of Lymphatic Enlargement of the Right Leg and Thigh. By W. H. Day, M. D. ... 572
Case of Puerperal Convulsions Attended with Rupture of the Uterus, and Recovery. By A. C. Rankin, M. D.... 581

Clinical Reports.
Notes from Private Practice:
Is the Bite of the Heterodon, or Spreading Adder, Venomous?......... 585
Case of Symmetrical Gangrene of the Fingers... 587
Case of Traumatic Tetanus............... 589
Case of Alopecia... 592
Three Cases of Molluscum Contagiosum... 593

Society Reports:
Transactions of the Chicago Gynæcological Society... 596
Michigan State Board of Health........... 604

Foreign Correspondence:
Clinical Reports from Prague and Berlin. 608

Editorial:
Valedictory of the Year... 616
To our Subscribers... 618

Reviews and Book Notices... 619
Books and Pamphlets Received............. 633

Selection:

The Critical Period of Homœopathy...... 636

Summary:
*Practical Medicine:*
The Etiology of Typhoid Fever......... 643
The Nature of Sciatica... 644

*Surgery:*
Foreign Body in the Rectum—Successful Removal ... 646
Action of Parenchymatous Injection of Glacial Acetic Acid on Carcinoma. 647
Treatment of Burns... 647

*Obstetrics:*
A New Device for Arresting Post Partum Hæmorrhage... 647

*Ophthalmology:*
Retinal Photo-Chemistry... 648

*Pathology:*
Renal Calculus Largely Composed of Indigo... 650
On Excision of the Primitive Sclerosis of Syphilis... 651

*Therapeutics:*
Therapeutics of Diphtheria During the Year 1877... 652
Concerning the Administration of Quinine... 656
Codeia in Cancer of the Pylorus......... 657
Heinsch's Sugar Test for Dangerous Organic Matter in Water.............. 657
Ammonio-Sulphate of Copper in Epileptiform Facial Neuralgia... 657
Warts... 657

*New Instruments:*
For Rapid and Forcible Dilatation of Cervical Canal for Removal of Offending Bodies... 658
Flexible Metallic Uterine Sound and Flexible Metallic Probe... 661

Items.....................584, 595, 615, 635, 642, 663

Obituary... 670

Announcements for the Month............. 672

Notice to Contributors... v advts.

